# Ambient Temperature and Morbidity: A Review of Epidemiological Evidence

**DOI:** 10.1289/ehp.1003198

**Published:** 2011-08-08

**Authors:** Xiaofang Ye, Rodney Wolff, Weiwei Yu, Pavla Vaneckova, Xiaochuan Pan, Shilu Tong

**Affiliations:** 1 School of Public Health and Institute of Health and Biomedical Innovation, and; 2 Mathematical Sciences Discipline, Faculty of Science and Technology, Queensland University of Technology, Brisbane, Queensland, Australia; 3 Department of Occupational and Environmental Health, Peking University School of Public Health, Beijing, China

**Keywords:** climate change, heat wave, hospital admission, morbidity, review, temperature

## Abstract

Objective: In this paper, we review the epidemiological evidence on the relationship between ambient temperature and morbidity. We assessed the methodological issues in previous studies and proposed future research directions.

Data sources and data extraction: We searched the PubMed database for epidemiological studies on ambient temperature and morbidity of noncommunicable diseases published in refereed English journals before 30 June 2010. Forty relevant studies were identified. Of these, 24 examined the relationship between ambient temperature and morbidity, 15 investigated the short-term effects of heat wave on morbidity, and 1 assessed both temperature and heat wave effects.

Data synthesis: Descriptive and time-series studies were the two main research designs used to investigate the temperature–morbidity relationship. Measurements of temperature exposure and health outcomes used in these studies differed widely. The majority of studies reported a significant relationship between ambient temperature and total or cause-specific morbidities. However, there were some inconsistencies in the direction and magnitude of nonlinear lag effects. The lag effect of hot temperature on morbidity was shorter (several days) compared with that of cold temperature (up to a few weeks). The temperature–morbidity relationship may be confounded or modified by sociodemographic factors and air pollution.

Conclusions: There is a significant short-term effect of ambient temperature on total and cause-specific morbidities. However, further research is needed to determine an appropriate temperature measure, consider a diverse range of morbidities, and to use consistent methodology to make different studies more comparable.

It is widely accepted that climate change is occurring and that it is caused mainly by increased emissions of anthropogenic greenhouse gases, particularly over the last few decades [Intergovernmental Panel on Climate Change (IPCC) 2007a]. Global mean temperature increased by 0.07°C per decade between 1906 and 2005, compared with 0.13°C per decade from 1956 to 2005 (IPCC 2007b). Not only has the average global surface temperature increased, but the frequency and intensity of temperature extremes have also changed [IPCC 2007a; World Health Organization (WHO) 2008]. Heat wave episodes have been associated with significant health impacts, for example, in 1995 in Chicago, Illinois (Semenza et al. 1999), in 2003 in Europe (Cerutti et al. 2006; Johnson et al. 2005; Larrieu et al. 2008; Mastrangelo et al. 2007; Oberlin et al. 2010), in 2006 in California (Knowlton et al. 2009), and in 2009 in southeastern Australia (National Climate Centre 2009). In addition, episodes of extreme cold (cold spells) are a concern in high-latitude regions (Pattenden et al. 2003) such as Russia (Revich and Shaposhnikov 2008), the Czech Republic (Kysely et al. 2009), and the Netherlands (Huynen et al. 2001).

The effect of ambient temperature on morbidity is a significant public health issue. Every year, a large number of hospitalizations are associated with exposure to extreme ambient temperatures, especially during heat waves and cold spells (Juopperi et al. 2002; Michelozzi et al. 2009; Schwartz et al. 2004; Semenza et al. 1999). For example, during the 1995 Chicago heat wave, it was estimated that there were 1,072 (11%) excess hospital admissions among all age groups, including 838 (35%) among those 65 years of age and older, with dehydration, heat stroke, and heat exhaustion as the main causes (Semenza et al. 1999). Actual numbers of morbidities may be greater than reported, because heat- or cold-related conditions may be listed as secondary diagnoses, and many studies have often considered primary diagnoses only (Kilbourne 1999; Semenza et al. 1999). Both heat- and cold-related morbidities occur more frequently among the elderly, as they are more vulnerable to temperature changes (Johnson et al. 2005; Knowlton et al. 2009; Kovats et al. 2004; Panagiotakos et al. 2004). In addition, urban residents may be exposed to higher temperatures than residents of surrounding suburban and rural areas because of the “heat island effect” resulting from high thermal absorption by dark paved surfaces and buildings, heat emitted from vehicles and air conditioners, lack of vegetation and trees, and poor ventilation (Barry and Chorley 2003; Hajat and Kosatsky 2009; O’Neill and Ebi 2009). Because of the urban heat island effect, people in urban areas are usually at an increased risk of morbidity from ambient heat exposure (O’Neill and Ebi 2009). The morbidity effect of temperature is likely to become more severe as the number of elderly people increases (from 737 million persons > 60 years old in 2009 to 2 billion by 2050 globally) and the proportion of urban residents increases (by approximately 18% over the next 40 years) and because climate change will continue for at least the next several decades, even under the most optimistic scenarios [IPCC 2007a; United Nations Department of Economic and Social Affairs (UNDESA) 2010a, 2010b].

In this paper, we assess the current epidemiological evidence concerning the effects of temperature on morbidity, identify knowledge gaps in this field, and make recommendations for future research directions.

## Methods

The PubMed electronic database was used to retrieve published studies examining the relationship between ambient temperature and morbidity of noncommunicable diseases (we excluded communicable diseases such as vector-borne diseases, as the research designs and analysis methods differ between communicable and noncommunicable diseases). Our primary search used the following U.S. National Library of Medicine Medical Subject Headings (MeSH terms) and key words: weather, climate, temperature, morbidity, hospitalization, emergency medical services, family practice, primary health care, heat wave, cold surge, and cold spell. All subterms were included, and we limited the search to original epidemiological studies published in English before 30 June 2010.

To examine the relationship between ambient temperature and morbidity, all relevant studies were included in this review. Eligibility included any epidemiological studies that used original data and appropriate effect estimates [e.g., regression coefficient, relative risk (RR), odds ratio (OR), percent change in morbidity, and morbidity or excess morbidity after heat waves]; where ambient temperature or a composite temperature measure was a main exposure of interest; and where the outcome measure included a noncommunicable disease (e.g., cardiovascular, cerebrovascular, or respiratory diseases). Titles and abstracts were screened for relevance, and full texts were then obtained for further assessment if papers met the inclusion criteria. We also inspected the reference list of each article to check if any studies were missed from the primary electronic search.

## Results

**Figure 1:**
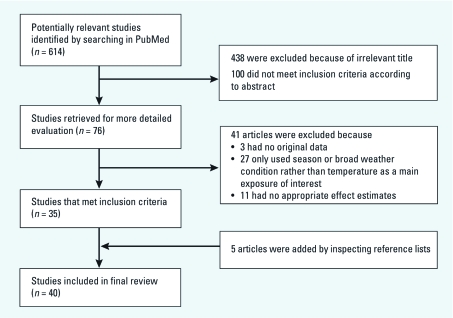
Flow chart of literature search strategy.

A total of 614 articles were identified from the PubMed (National Library of Medicine 2010) database, and 76 initially met the eligibility criteria for full-text inspection after reading the abstracts (Figure 1). We excluded 41 articles because 3 had no original data, 27 assessed only the effect of season or broad weather conditions, and 11 did not report appropriate effect estimates. Five studies were added after manually inspecting the reference lists of all relevant articles. Finally, 40 articles were included in the review. Of these, 24 examined the relationship between general ambient temperature and morbidity, 15 investigated short-term effects of heat waves on morbidity, and 1 assessed both general ambient temperature-related and heat wave–related health effects.

## Methodological Considerations

*Study designs and statistical approaches.* A variety of study designs were used to assess the health effects of heat waves and cold spells and to characterize the association between temperature and morbidity. Most studies employed either a descriptive or time-series study design. Statistical methods varied with study design.

*Descriptive studies.* Simple comparisons were applied in the analysis of health effects of isolated heat waves in seven studies (Cerutti et al. 2006; Ellis et al. 1980; Johnson et al. 2005; Jones et al. 1982; Knowlton et al. 2009; Rydman et al. 1999; Semenza et al. 1999) in addition to studies where risk factors and illnesses studied during heat waves and cold spells were often characterized in details. To assess effects of heat waves on morbidity, most of the studies estimated an excess proportion by comparing observed versus expected morbidity. Many methods were used to calculate expected morbidity, which largely depended on the chosen baseline. Usually, expected hospital admissions were based on the average number of admissions during comparison days or weeks, for example, the days prior to or after a heat wave, or the same time period in previous years without heat waves (Huynen et al. 2001; Johnson et al. 2005; Semenza et al. 1999; Yang et al. 2009). Although such comparative analyses can provide useful insights into the short-term response of the population to a heat wave or cold spell event, they may underestimate or overestimate effects because of the use of an inappropriate baseline, potential morbidity displacement, and lack of control for confounding factors (e.g., air pollution).

*Time-series studies.* Time-series studies have been widely used to examine short-term effects of temperature on morbidity (Kovats et al. 2004; Linares and Diaz 2008; Michelozzi et al. 2009; Schwartz et al. 2004). Morbidity counts or rates were usually used as the outcome measures, whereas temperature measurements at corresponding intervals were employed as exposure indicators. Time-series analysis using daily data was commonly applied, but weekly or monthly data were used in some studies, which may make it difficult to detect acute temperature effects on morbidity (Roger and Francesca 2008; Touloumi et al. 2004). Effects were often estimated as the percent change in morbidity per unit increase (or decrease) in temperature (e.g., one or several degrees Centigrade or interquartile range change) (Ebi et al. 2004; Green et al. 2009; Koken et al. 2003; Lin et al. 2009). In this design, confounding is limited to time-varying factors such as air pollution, influenza epidemics, season, holiday (e.g., Christmas, New Year), and the day of the week (which could be taken into account in multivariable models).

In general, both hot and cold extremes of temperature have an adverse effect on health, which suggests a potential nonlinearity of the temperature effect. Thus, Poisson regression through generalized additive models (GAM) was widely used to assess the temperature–morbidity relationship after adjustment for long-term effects, seasonality, and other seasonally varying factors (Barnett et al. 2005; Ren et al. 2006; Schwartz et al. 2004). Alternatively, analyses were stratified by summer/winter or warm/cold periods to remove seasonal patterns and simplify analyses (Lin et al. 2009; Michelozzi et al. 2009; Piver et al. 1999; Wang et al. 2009; Ye et al. 2001). Appropriate temperature thresholds were selected based on model fit (Kovats et al. 2004) or selected cutoff (e.g., percentiles or absolute values of the temperature distribution) (Michelozzi et al. 2009), which facilitated the analysis of health effects of temperature extremes.

*Exposure measurements.* Mean daily temperature (Kovats et al. 2004; Liang et al. 2008; Schwartz et al. 2004) was a simple and common temperature indicator. Minimum (Ebi et al. 2004; Linares and Diaz 2008) and maximum temperatures (Linares and Diaz 2008; Wang et al. 2009) were also used in many studies. Diurnal temperature range was reported to be a risk factor for patients suffering from cardiovascular and respiratory diseases (Liang et al. 2008, 2009). Other studies used biometeorological indices such as apparent temperature (Green et al. 2009; Michelozzi et al. 2009) and Humidex (Mastrangelo et al. 2007). These perceived indices combine air temperature and humidity and are considered to be better measures of the effect of temperature on the human body than is temperature alone. However, no single temperature measure was reported to be superior to the others to predict the mortality (Barnett et al. 2010).

In examining the effect of heat waves (and cold spells), the first thing to be considered is the definition of the exposure, which may vary with geographic location and climatic condition because the sensitivity of populations to heat stress varies geographically (Hansen et al. 2008a; Knowlton et al. 2009; Kovats et al. 2004; Revich and Shaposhnikov 2008; Robinson 2001). As heat effects in one area may not be applicable to another area, multicity studies were recently conducted to assess general heat effects (Anderson and Bell 2009; Green et al. 2009; Michelozzi et al. 2009). Besides heat wave intensity, heat wave duration is also an important risk factor in estimating the health effect of heat episodes (Mastrangelo et al. 2007). Vulnerability to heat stress depends on many factors, such as age, preexisting diseases, environmental humidity, and adaptative response (Bouchama and Knochel 2002; Cui et al. 2005; Parsons 2003). A long heat wave could lead to accumulated heat stress on the body when heat produced and obtained from the environment overwhelms the heat loss by thermoregulation. Over consecutive hot days without cooler nights, individuals may suffer from thermoregulatory failure, increasing the risk of illnesses (Bouchama and Knochel 2002; Parsons 2003). There is also evidence that the effect of extreme cold might increase with increasing duration, as low temperature can lead to cardiovascular stress by increasing platelet counts, red cells, blood viscosity, plasma cholesterol, fibrinogen, and blood pressure and increase susceptibility to pulmonary diseases by causing bronchoconstriction (Hong et al. 2003; Huynen et al. 2001; Keatinge et al. 1984; Mercer 2003).

*Outcome measurements.* Although admissions for some heat-related conditions such as heat stroke, heat exhaustion, fluid and electrolyte abnormalities, and acute renal failure were higher during heat waves (Hansen et al. 2008b; Knowlton et al. 2009; Semenza et al. 1999), actual numbers were assumed to be underestimated, as many cases were likely to be coded cardiovascular or respiratory diseases in primary diagnoses. As a result, some researchers recommend that primary and secondary discharge diagnoses be considered together to reduce misclassification of heat-related diseases (Kilbourne 1999; Semenza et al. 1999). The common causes of morbidity evaluated in previous studies included total cardiovascular and respiratory diseases (Lin et al. 2009; Linares and Diaz 2008; Michelozzi et al. 2009; Ren et al. 2006) and specific diseases such as stroke (Kyobutungi et al. 2005; Ohshige et al. 2006; Wang et al. 2009), acute myocardial infarction (Chang et al. 2004; Ebi et al. 2004; Schwartz et al. 2004; Ye et al. 2001), and acute coronary syndrome (ACS; Liang et al. 2008; Panagiotakos et al. 2004).

Some direct cold injuries occur during winter, such as frostbite and hypothermia (Hassi et al. 2005; Juopperi et al. 2002). Ischemic stoke (Hong et al. 2003), coronary events (Barnett et al. 2005), and cardiovascular and respiratory diseases (Hajat et al. 2004; Hajat and Haines 2002) were reported in the studies of cold temperature morbidity. No study has investigated the morbidity after a cold spell, whereas only a few studies examined cardiovascular and respiratory mortality of extreme cold temperatures (Huynen et al. 2001; Kysely et al. 2009; Revich and Shaposhnikov 2008).

## Major Findings

A number of studies examined the relationship between ambient temperature and morbidity. These studies identified the general risks of temperature as well as temperature extremes in multiple areas over time, using different research designs. Table 1 summarizes the findings of ambient temperature–morbidity studies, whereas Table 2 summarizes the findings of heat wave studies.

**Table 1:** Characteristics of the ambient temperature–morbidity studies (*n* = 25).

Study	Location and time	Main temperature exposure variable	Outcome	Research design and statistical analysis	Key findings	Comments
Studies of both hot and cold exposure												
Ebi et al. 2004		Three U.S California regions; 1983–1997 and January–June 1998		Minimum and maximum temperature		Hospitalizations for AMI, angina pectoris, CHF, stroke		Time-series; Poisson regression, GEE		Temperature changes (3°C increase in maximum temperature or 3°C decrease in minimum temperature) increased hospitalizations for residents ≥ 70 years of age by 6–13% in San Francisco and by 6–18% in Sacramento; small changes in Los Angeles		Normal weather periods and El Niño events were analyzed separately and combined; no air pollution was controlled for
										Association varied by region, age, and sex		
										Lag: 7 days		
Schwartz et al. 2004		Twelve U.S. cities; 1986–1994		Daily mean temperature		Urgent hospital admissions for heart disease and MI, ≥ 65 years of age		Time-series; Poisson regression, distributed lag models		Positive linear relation for all heart diseases		Systematically examined temperature and morbidity in several U.S. cities with various climates; air pollution was not controlled for as confounder
										RR = 1.15 (0.96, 1.37) increased risk of 80°F (compared with 0°F)		
										Harvesting effect (within 10 days) in hot temperatures but not in cold weather		
										Similar but smaller effects of temperature for MI admissions		
										Lag: 0, 1 day		
Bayentin et al. 2010		Quebec, Canada; 1 April 1989–31 March 2006		Mean temperature		Hospitalization for IHD		Time-series; GAM		V- or U-shaped curves		No air pollution was controlled for; only description of deprivation indexes presented, rather incorporated it into the model
										Threshold different for each region and for both sexes		
										Lag duration dependent on the region		
										High admissions observed earlier among adults in the ≥ 65 year age group; high excess risks associated with high smoking prevalence and high deprivation indexes (material or social)		
Ohshige et al. 2006		Yokohama, Japan; 1992–2003		Mean temperature		Stroke incidence of emergency transport events, ≥ 50 years of age		Time-series; Poisson regression, ordinary least squares regression		Significant negative effect of mean temperature on the stoke incidence of the emergency transport events		Ranges rather than actual values of temperature, humidity and barometric pressure were used; no air pollution was controlled for
	
Liang et al. 2008		Taichung, Taiwan; 1 January 2000–​31 March 2003		Mean temperature, DTR		Emergency room admissions for ACS		Time-series; Poisson regression		28.4% increase risk for 17–27°C and 53.9% for < 17°C (reference 27–29°C of mean temperature)		Only one hospital was included
										34.4% increase risk for > 9.6°C (reference < 5.8°C of DTR)		
Liang et al. 2009		Taichung, Taiwan; 2001–2002		Mean temperature, DTR		Emergency room admissions for COPD		Time-series; Poisson regression		RR = 1.2 for 22.95–26.58°C and RR = 1.5 for < 22.95°C (reference 29.42°C of mean temperature)		Only one hospital was included
										RR = 1.14 for > 9.6°C (reference < 6.6°C of DTR)		
Ren et al. 2006		Brisbane, Australia; 1996–2001		Minimum temperature		Hospital admissions and emergency visits for CVD and RD		Time-series; Poisson GAM, nonparametric bivariate response model, nonstratification model		PM_10_ modified the effects of temperature on respiratory and cardiovascular hospital admissions with enhanced adverse effects at high level, but no clear evidence for emergency visits		First to examine the PM_10_ modification of the association between temperature and health outcomes
										Lag: 0–2 days		
Wang et al. 2009		Brisbane, Australia; summer and winter, 1996–2005		Minimum and maximum temperature		Emergency admissions for PIH and IS		Time-series; GEE		Different response of PIH and IS to temperature variation by season		First to examine the impact of temperature variation on different types of stroke morbidity in a subtropical city
										1°C increase in minimum and maximum temperature 15% (5–26%) and 12% (2–22%) for PIH among adults < 65 years of age in summer; in winter, 1°C decrease in minimum and maximum temperature 6% (2–10%) and 7% (4–11%) for PIH among those ≥ 65 years of age		
Rothwell et al. 1996		Oxfordshire, United Kingdom; 1980s		Mean temperature		First ever in a lifetime stroke		Chi-square		No significant seasonal variation was reported. The incidence of primary intracerebral hemorrhage was increased at low temperature, but not for ischemic stroke or subarachnoid hemorrhage		Community study rather than hospital-based study was conducted to avoid selection bias; the incidence of first ever in a lifetime stroke was collected; no confounders were controlled for
Panagiotakos et al. 2004		Athens, Greece; January 2001–August 2002		Daily mean and minimum and maximum temperature, THI		Nonfatal ACS in the emergency units		Time-series; GAM		Negative correlation between hospital admissions for ACS and daily temperature		No air pollution was controlled for
										1°C decrease in mean temperature was associated with a 5.0% (4.6–5.4%) increase in hospital admissions for ACS; similar results for minimum and maximum temperatures and for THI. Stronger association for females and the elderly		
Kyobutungi et al. 2005		Heidelberg, Germany; August 1998–January 2000		Maximum temperature and 24-hr difference in maximum temperature		IS incidence		Case-crossover; conditional logistic regression		No risk associated with ambient maximum temperature and its 24-hr difference		Used both absolute temperature and temperature difference in one day; no air pollution was controlled for
*continued next page*												
Table 1. continued.												
Study		Location and time		Main temperature exposure variable		Outcome		Research design and statistical analysis		Key findings		Comments
Dawson et al. 2008		Scotland; 1 May 1990–​22 June 2005		Mean and minimum and maximum temperature, mean temperature change over the preceding 24 and 48 hr		Hospital admissions for acute stroke		Time-series; negative binomial regression, Poisson regression		1°C increase in mean temperature during the preceding 24 hr 2.1% (0.7–3.5%) increase in ischemic stroke admissions		No air pollution was controlled for
Chang et al. 2004		Seventeen countries worldwide (including Africa, Asia, Europe, and Latin America), February 1989–January 1995		Monthly mean temperature		Monthly number of newly diagnosed cases of VTE, stroke, or AMI, women 15–49 years of age		Time-series; negative binomial regression		Significant negative associations with temperature for stroke and AMI, but not for VTE		Monthly mean values were used; no air pollution was controlled for
										5°C increase in mean temperature IRR = 0.93 (0.89, 0.97) for stroke and IRR = 0.88 (0.80, 0.97) for AMI		
										Lag: within 1 month		
										No modification of age and high blood pressure		
Hot exposure only												
Koken et al. 2003		Denver, CO, United States; July–August, 1993–1997		Maximum temperature		Hospital admissions for CVD, > 65 years of age		Time-series; Poisson regression, GLM, GEE		1°C increase 17.5% (2.9 to 34.3%), 13.2% (2.9–24.4%), –12.5% (–18.9 to –5.5%), and –28.3% (–38.4 to –16.5%) for AMI, CHF, coronary atherosclerosis, and pulmonary heart disease, respectively		Only July and August were included
										Lag: 0, 1 day		
										Male had higher numbers of hospital admissions than female		
Green et al. 2009		Nine U.S. California counties; May–September, 1999–2005		Mean apparent temperature		Hospital admissions for CVD, RD, diabetes, dehydration, heat stroke, intestinal infectious diseases, and ARF		Case-crossover; conditional logistic regression, meta-analysis		Per 10°F increase apparent temperature, 2.0% (0.7–3.2%) excess risk in RD, 3.7% pneumonia, 3.1% diabetes, 10.8% dehydration, 7.4% ARF, 404.0% heat stroke, and –10.4% in hemorrhagic stroke		GIS methods were used to improve exposure assessment
										Lag: 0		
										Effect differed by age, little evidence of effect modification of sex, ethnicity, PM_2.5_, ozone, and nonlinearity		
Lin et al. 2009		New York, United States; summer, 1991–2004		Mean temperature, mean apparent temperature, 3-day moving average of apparent temperature		Hospital admissions for CVD and RD		Time-series; GAM, linear-threshold model		1°C increase above mean temperature threshold 2.7% (1.25–4.16%) for RD on the same day and 3.6% (0.32–6.94%) for CD on lag-3 day		One city was included; first to examine the independent and joint effects of temperature and humidity; conducted stratified analyses based on family income
										1°C increase above mean apparent temperature threshold 2.1% (1.1–3.1%) and 1.4% (0.4–2.4%) for RD on the same day and 1 day later; 2.5%, 2.1%, and 3.6% at 1, 2, and 3 days later, respectively, for CD		
										Lag: 0–3 days		
										Positive interaction between high temperature (> 29.4°C) and humidity		
										Greater increases of CVD and RD admissions in Hispanic persons, the elderly, and low-income persons; sex and disease type interacted with temperature		
Piver et al. 1999		Tokyo, Japan; July and August 1980–1995		Daily maximum temperature		Emergency transport cases for heat stroke		Time-series; GLM, GEE		Daily maximum temperature associated with heat stroke		Only July and August were included
										Greater number of heat stroke emergency transport cases for males than for females; smallest risk for females 0–14 years of age and the greatest risk for males > 65 years of age		
Ye et al. 2001		Tokyo, Japan; July and August 1980–1995		Daily maximum temperature		Hospital emergency transports for CVD and RD > 65 years of age		Time-series; GLM, GEE		Except hypertension and pneumonia, daily maximum temperature not associated with hospital emergency transport		Only July and August were included. Several specific diseases were considered
										1°C increase 3.8% (2.0–5.0%) increase in pneumonia and 1.4% (0.4–2.0%) decrease in hypertension		
										Lag: 0		
Kovats et al. 2004		Greater London, United Kingdom; 1 April 1994–31 March 2000		Three-day moving average temperature		Emergency hospital admissions for CVD, RD, CD, renal disease, ARF, calculus of the kidney and ureter		Time-series; autoregressive Poisson regression, hockey-stick model		No relation between total emergency hospital admissions and high temperature; 1°C above threshold 5.44% (1.92–9.09%) for RD, 1.30% (0.27–2.35%) for renal disease, 0.24% (0.02–0.46%) for children < 5 years of age, and 10.86% (4.44–17.67%) for RD for adults in the ≥ 75 age group		Contrasting patterns of mortality and hospital admissions during hot weather
*continued next page*												
Table 1. continued.												
Study		Location and time		Main temperature exposure variable		Outcome		Research design and statistical analysis		Key findings		Comments
Linares and Diaz 2008		Madrid, Spain; May–September, 1995–2000		Maximum and minimum temperatures		Emergency hospital admissions for all causes, RD, and CVD		Time-series; ARIMA		V-shaped relationship		Data from one hospital were used
										1°C increase above maximum temperature threshold 36°C 4.6% (0.9–8.4%) for all causes in all age groups (lag 0), 17.9% (9.5–26.0%) for all causes among adults in the ≥ 75 year age group (lag 1), and 27.5% (13.3–41.4%) for RD among adults in the ≥ 75 year age group (lag 0); no relationship between heat (> 36°C) and admissions for CVD in all the age groups		
										Lag: 0, 1		
Michelozzi et al. 2009		Twelve European cities; April–September, each city ≥ 3 years during 1990–2001		Maximum apparent temperature		Hospital admission for CVD, CD, and RD		Time-series; GEE, random effect meta-analysis		No or tendentious negative relationship between temperature and CVD and CD; 1°C increase above threshold 14.5% (1.9–7.3%) in Mediterranean and 13.1% (0.8–5.5%) in North-Continental region among adults in the ≥ 75 year age group for RD, almost twice that for all ages		First attempt to evaluate the effect of temperature on several morbidity outcomes using a standardized methodology in a multicenter European study
										Lag: 0–3 days		
Cold exposure only												
Hong et al. 2003		Incheon, Korea; 1998–2000		Daily average temperature, 3-hr average temperature		IS onset		Case-crossover; conditional logistic regression		IS onset was associated with decrease in temperature. One interquartile range decrease in temperature (17.4°C) OR = 2.9 (1.5–5.3) for IS on lag 1		Used bidirectional control selection scheme; assessed lag structure in hours
										Lag: 1 day, 24–54 h		
										Stronger effects in winter and for women, adults > 65 years of age, nonobese persons, and those with hypertension or hypercholesterolemia		
Hajat and Haines 2002		London, United Kingdom; January 1992–September 1995		Mean temperature		GP consultation for RD and CVD, adults ≥ 65 years of age		Time-series; GAM		1°C decrease < 5°C, 10.5% (7.6–13.4%) increase in RD and 12.4% (0.7–25.4%) in asthma; no relationship between cold temperature and GP for CVD		Primary care data could be influenced by patient behaviors and service availability (i.e., the time when a patient can be seen by a general practitioner; access to convenient medical facilities)
										Lag: 6–15 days		
Hajat et al. 2004		United Kingdom; 1992–2001		Mean temperature		GP consultations for RD, adults ≥ 65 years of age		Time-series; GLM		Linear association between low temperature and an increase in RD in all 16 locations		Primary care data were used
										1°C decrease < 5°C, biggest effect 19.0% (13.6–24.7%) increase in Norwich for lower respiratory tract infections; weaker relationships for upper respiratory tract infections consultation		
										Lag: 0–20 days		
										Larger effects in the north than in the south		
Barnett et al. 2005		Twenty-four populations worldwide, 1980–1995		Mean temperature		Daily records of coronary events, persons 35–64 years of age		Time-series; distributed lag model, hierarchical meta-regression; logistic model, Bayesian hierarchical model		Daily rates of coronary events negatively correlated with the average temperature		Air pollutants and respiratory infections were not controlled for
										Lag: 0–3 days		
										Coronary event rates increased more in populations living in warm climates than in cold climates		
										Greater increase for women than for men with the odds 1.07 (1.03, 1.11)		
Abbreviations: ACS, acute coronary syndrome; AMI, acute myocardial infarction; ARF, acute renal failure; ARIMA, autoregressive integrated moving average model; CD, cerebrovascular diseases; CHF, congestive heart failure; COPD, chronic obstructive pulmonary disease; CVD, cardiovascular diseases; DTR, diurnal temperature range; GAM, generalized additive models; GEE, generalized estimating equations; GIS, geographic information system; GLM, generalized linear models; GP, general practitioner; IHD, ischemic heart disease; IRR, incidence rate ratio; IS, ischemic stroke; OR, odds ratio; MI, myocardial infarction; PIH, primary intracerebral hemorrhage; PM_10_, particulate matter < 10 µm in aerodynamic diameter; RD, respiratory diseases; RR, relative risk; THI, thermo-hydrological index; VTE, venous thromboembolism.												

**Table 2:** Characteristics of the heat wave–morbidity studies (*n* = 16).

Study	Location and time	Main temperature exposure variable	Outcome	Research design and statistical analysis	Key findings	Comments
Ellis et al. 1980		Birmingham, United Kingdom; 24 June–​8 July 1976		2-week heat wave with the reference period (2-week periods before and after the heat wave, same days in 1974 and 1975)		Mortality and morbidity		Descriptive study		Daily deaths increased significantly during heat wave. No increase of new claims for sickness benefit among working people. More hospital admissions during heat wave than for the same period in 1975 or 1974. Modest increase in the episodes of sickness in two large general practices.		One single heat wave was studied. Four different types of morbidity were used.
Applegate et al. 1981		Memphis, TN, United States; 25 June–20 July 1980		Heat wave		Heat-related emergency room visits, hospital admissions, and deaths		Descriptive study		Heat-related emergency room visits, hospital admissions, and deaths rose markedly during heat wave. The most severe effects were seen among elderly, poor, black, inner-city residents.		A survey of elderly persons receiving home health care was conducted during the heat wave.
Jones et al. 1982		St Louis, MO, and Kansas, United States; June and July 1980		Heat wave with the same periods in 1979 and 1978		Total hospital admissions, emergency room visits, and deaths from all causes		Descriptive study		Deaths, hospital admissions, and emergency room visits from all causes increased during heat wave in 1980 compared with 1979 and 1978 in St Louis and Kansas. Higher heat stroke rates were found among the elderly, the poor, and nonwhites.		Hospital records, medical examiners’ records, and death certificates were used to identify cases.
Faunt et al. 1995		Adelaide, Australia; February 1993		10-day heat wave		Emergency department presentations		Retrospective survey; descriptive analysis		Ninety-four patients had heat-related illness; of these, 78% had heat exhaustion, 85% were ≥ 60 years of age, 20% came from institutional care, 48% lived alone, and 30% had poor mobility. Severity was related to preexisting conditions.		One single heat wave was studied. Only four hospitals were included.
Rydman et al. 1999		Chicago, IL, United States; 6–19 July 1995		Heat wave with the same period in 1994		Emergency department visits		Descriptive study; chi-square, *t*-test, linear regression		There were 2,446 excess morbidity cases. Heat morbidity increased 5 days before the first heat-related death. The most frequent heat-related diagnoses were hyperthermia, heat exhaustion, and heat stroke. Different morbidity was found in age groups, comorbid primary diseases, and disposition.		One single heat wave was studied.
Semenza et al. 1999		Chicago, IL, United States; 13–19 July 1995		Heat-wave week with four non–heat wave comparison weeks		Excess hospital admissions		Descriptive study		1,072 (11%) more hospitalizations and 838 (35%) among patients ≥ 65 years of age—most of these were due to dehydration, heat stroke, heat exhaustion, and ARF. There was significant excess of underlying CVDs, diabetes, renal diseases, and nervous system disorders.		Different spectrum of illnesses between primary and all discharge diagnoses during the heat wave.
Kovats et al. 2004		Greater London, United Kingdom; 29 July–3 August 1995		Heat wave		Excess emergency hospital admissions		Time-series; autoregressive Poisson regression, hockey-stick model		Hospital admissions showed a small nonsignificant increase of 2.6% (95% CI: –2.2, 7.6), whereas daily mortality rose by 10.8% (95% CI: 2.8, 19.3).		Contract between hospital admissions and mortality.
Johnson et al. 2005		England; 4–13 August 2003		10-day heat wave period compared with the same time in 1998–2002		Excess mortality and emergency hospital admissions		Descriptive study		There were 2,091 excess deaths (17%). People ≥ 75 years of age were at the greatest risk. An excess of only 1% in total emergency hospital admissions was found.		The increases of emergency hospital admissions were not comparable with mortality.
Cerutti et al. 2006		Ticino, Switzerland; 2003		Three heat waves compared with previous years (2000–2002)		Excess mortality and emergency ambulance service intervention		Descriptive study		The 2003 mortality in the population was not significantly different from previous years except for the first heat wave. The number of ambulance service interventions was larger than during the previous years.		Daily rates were used rather than raw numbers of deaths or interventions.
Mastrangelo et al. 2007		Veneto Region, Italy; 1 June–31 August 2002–2003		Five consecutive heat waves		Daily count of hospital admission by cause among people ≥ 74 years of age		Ecologic study; GEE		Heat wave duration, not intensity, increased the risk of hospital admissions for heart diseases and RD 16% (*p* < 0.0001) and 5% (*p* < 0.0001), respectively, with each additional day of heat wave duration. At least 4 consecutive hot, humid days were required to observe a major increase in hospital admissions. Hospital admissions peaked equally at the first and last heat wave in 2003.		Heat wave duration, intensity, and timing were considered.
Nitschke et al. 2007		Adelaide, Australia; July 1993–June 2006		Thirty-one heat waves compared with non–heat wave periods during spring and summer		Daily ambulance transports, hospital admissions, and mortality		Case-series study; Poisson regression, negative binomial regression		Total ambulance transport and total hospital admissions increased by 4% (95% CI: 1, 7) and 7% (95% CI: –1, 16), respectively. Admissions for mental health, renal diseases and IHD among people 65–74 years of age increased by 7% (95% CI: 1, 13), 13% (95% CI: 3, 25), and 8% (95% CI: 1, 15), respectively. Mortality did not increase.		Three kinds of health end points were used.
Hansen et al. 2008a		Adelaide, Australia; 1 July 1993–​30 June 2006		Heat waves, daily maximum temperature		Daily counts of admissions and MBDs		Time-series; Poisson regression, hockey-stick regression		Hospital admissions increased by 7.3% during heat waves. Above a threshold of 26.7°C, there was a positive association between ambient temperature and hospital admissions for MBDs. MBDs mortalities increased during heat waves in the elderly.		First to characterize specific disorders that contributed to increased psychiatric morbidity and mortality during heat waves.
*continued next page*												
Table 2. continued.												
Study		Location and time		Main temperature exposure variable		Outcome		Research design and statistical analysis		Key findings		Comments
Hansen et al. 2008b		Adelaide, Australia; 1995–2006		Heat waves		Daily hospital admissions for renal disease, ARF, and renal dialysis		Time-series; Poisson regression		Admissions for renal disease and ARF increased during heat waves, with IRR = 1.10 (95% CI: 1.00, 1.21) and IRR = 1.26 (95% CI: 1.04, 1.52), respectively. Hospitalizations for dialysis showed no increase. Pre-existing diabetes did not increase the risk of renal admission.		First investigated the association between high temperature and renal morbidity in a temperate Australian region.
Larrieu et al. 2008		France; 2003		2003 heat wave		Felt morbidity, objective morbidity of elderly people		Cross-sectional study; chi-square, *t*-test, logistic regression		During the heat wave, 8.8% of the subjects felt a deterioration of heath, and 7.8% declared an objective morbid outcome. Many factors were associated with morbidity.		It was an exploratory study using a questionnaire to collect data from subjects.
Knowlton et al. 2009		California, United States; 15 July–1 August 2006		Heat wave with the reference period (8–14 July, 12–22 August 2006)		Excess hospitalizations and emergency department visits		Descriptive study		16,166 excess emergency department visits and 1,182 excess hospitalizations. Emergency department visits (RR = 6.30, 95% CI: 5.67, 7.01) and hospitalizations (RR = 10.15, 95% CI: 7.79, 13.43) for heat-related causes increased. There were significant increases for ARF, CVD, diabetes, electrolyte imbalance, and nephritis. The heat wave impact on morbidity varied across regions, race/ethnicity, and age groups. Children (0–4 years of age) and the elderly (≥ 65 years of age) were at greatest risk.		Principal and the first nine secondary diagnoses were included. Used both emergency department visits and hospitalization.
Oberlin et al. 2010		Toulouse, France; 1–31 August 2003		Heat wave		Emergency department admissions of patients > 65 years of age		Retrospective study; descriptive analysis		Forty-two (5.5%) patients had heat-related illness. They were more likely to live in institutional care rather than at home and had longer length of stay and higher death rate than non–heat-related illness.		Double-checked medical record to ascertain heat-related illness.
Abbreviations: ARF, acute renal failure; CVD, cardiovascular diseases; GEE, generalized estimating equations; IRR, incidence rate ratio; MBDs, mental and behavioral disorders; RD, respiratory diseases.												

*Threshold effects of temperature.* A nonlinear relationship between temperature and morbidity was evident across different studies that illustrated U-, V-, or J-shaped patterns (Kovats et al. 2004; Liang et al. 2008; Lin et al. 2009; Linares and Diaz 2008), with the minimum morbidity at a certain temperature or temperature range (threshold temperature) and increased morbidity below and above the threshold. However, few studies identified clear threshold temperatures based on model fit (Kovats et al. 2004; Lin et al. 2009).

There is some evidence that both hot and cold threshold temperature for morbidity vary by location. For example, in a study in New York City, hospital admissions for respiratory diseases increased at temperatures > 28.9°C (Lin et al. 2009). However, the threshold temperature of respiratory hospital admissions in London, United Kingdom, was lower (23°C) (Kovats et al. 2004), as the cooler summers resulted in lower acclimatization to high temperature. The cold threshold temperature also differed for each region in Quebec, Canada, in winter (Bayentin et al. 2010).

Different thresholds have also been identified for different diseases. A large increase in emergency hospital admissions was observed for respiratory diseases at temperatures > 23°C in Greater London, whereas admissions for renal diseases increased above a lower temperature of 18°C (Kovats et al. 2004).

*Magnitude of the effects of temperature and heat wave.* Consistent with expectations that the relation between temperature and morbidity will follow a V- or J-shaped curve, a study in Taiwan reported that emergency room admissions for acute ACS were lowest for temperatures of 27–29°C. Compared with this baseline range, ACS admissions were 28.4% higher for average daily temperatures in the range of 17–27°C (with a slight increase > 29°C) and 53.9% higher for temperatures < 17°C (Liang et al. 2008). To fully assess the shape of the association between temperature and morbidity, it is necessary to evaluate associations across the entire temperature range throughout a year. Studies focused on associations during hot or cold seasons only usually show a linear association of temperature with morbidity. For example, Lin et al. (2009) reported increased counts of cardiovascular [3.6%; 95% confidence interval (CI): 0.3, 6.9] and respiratory diseases (2.7%; 95% CI: 1.3, 4.2) with a 1°C increase in temperature during the summer in New York City, whereas a study in Brisbane reported a decreased risk of emergency admissions for primary intracerebral hemorrhage with a 1°C increase in minimum temperature (RR = 0.95; 95% CI: 0.91, 0.98) during the winter (Wang et al. 2009). In contrast, a study of 12 European cities revealed that the association between temperature and cardiovascular and cerebrovascular hospital admissions tended to be negatively linear but did not reach statistical significance during hot seasons (Michelozzi et al. 2009). However, some studies that evaluated associations over the entire year also reported evidence of linear versus J- or V-shaped associations (Panagiotakos et al. 2004; Schwartz et al. 2004). For example, in 12 U.S. cities, average temperature was positively related to hospital admissions for heart diseases among adults ≥ 65 years old (Schwartz et al. 2004). Cardiovascular, respiratory, and cerebrovascular diseases comprise many subtypes that might react to temperature in different ways (Dawson et al. 2008; Lin et al. 2009; Wang et al. 2009; Ye et al. 2001). For example, hemorrhage stroke and ischemic stroke hospital admissions, both of which would be classified as cerebrovascular diseases, showed opposite relationships to temperature increases in California (Green et al. 2009). Additionally, an interquartile range increase in maximum temperature during hot seasons in Denver, Colorado, was associated with a 12.5% and 28.3% decrease in risk of hospitalization for coronary atherosclerosis and pulmonary heart disease, respectively, compared with a 17.5% increase for acute myocardial infarction among the elderly (Koken et al. 2003). These results suggested that patients with chronic rather than acute cardiovascular conditions might avoid outdoor exposures during unfavorable weather, resulting in a null or negative association. Moreover, if appointments for mild diseases are postponed or cancelled during extremely hot or cold periods, the effect of temperature on morbidity might be underestimated.

Despite evidence of variation among specific diseases, increased overall morbidity has been consistently associated with heat waves. For example, during a Chicago, Illinois, heat wave in 1995, there were 838 (35%) more hospital admissions of the elderly (≥ 65 years old) compared with the average number of admissions during comparable weeks (Semenza et al. 1999). A total of 16,166 (3%) excess emergency department visits and 1,182 (1%) excess hospitalizations occurred in California during the 2006 heat wave (Knowlton et al. 2009). In England, the 2003 heat wave caused an excess of 1% total emergency hospital admissions (Johnson et al. 2005). In a study in Adelaide, Australia, Nitschke et al. (2007) reported a 4% and 7% increase in total ambulance transport and hospital admissions during heat waves, respectively, compared with non–heat wave periods.

*Lag structure of temperature.* Some studies explored temporal patterns (lag structure) of the association between exposure to temperature over previous days and health risk on a particular day. Various lag days were reported for the association of temperature with morbidity, ranging from the same day (Green et al. 2009) to 1 month (Chang et al. 2004), with shorter lags during warmer seasons and longer lags during cooler seasons (Barnett et al. 2005; Hajat and Haines 2002). In a study of 12 U.S. cities, Schwartz et al. (2004) also reported that associations with hot temperatures were more immediate than with cold temperatures. Most recent studies have reported short-term effects of high temperature on the same day and the 3 days after heat exposure (Green et al. 2009; Koken et al. 2003; Lin et al. 2009). For example, Lin et al. (2009) observed that the greatest number of hospital admissions for respiratory and cardiovascular diseases was 0–1 days and 1–3 days after increased temperatures (Lin et al. 2009). Seven-day lag was used to evaluate the effect of temperature on hospital admissions for several specific cardiovascular diseases (Ebi et al. 2004). One-month lag has also been reported by a study that evaluated average temperatures over a monthly period across several whole years (Chang et al. 2004), but it was not clear whether the effects would have been more immediate if daily data had been evaluated. Hajat and Haines (2002) found a strong association between consultations for respiratory disease and mean temperature < 5°C over a 10-day period (i.e., 6–15 days before the consultation) in London, which implied a later and longer lag for cold temperatures than hot ones.

*Harvesting effects of temperature.* Evidence of a harvesting effect (e.g., mortality displacement) has been documented by studies of heat-related mortality (Braga et al. 2002; Muggeo and Hajat 2009) that showed an immediate increase in mortality followed by reduced mortality among susceptible people, consistent with a temporal advance in deaths that would have occurred later in time in the absence of exposure to heat or cold. However, the impact of harvesting on morbidity has not been fully investigated, and short-, intermediate-, and long-term effects should be examined to determine the impact of harvesting. Schwartz et al. (2004) reported evidence of a short-term advance in emergency hospital admissions for heart diseases and myocardial infarction among people ≥ 65 years of age within a few days after high-temperature exposure, with a positive association on the day of admission followed by a period of lower-than-average admissions, returning to the baseline after a week. No evidence of a harvesting effect was observed for cold weather in this study (Schwartz et al. 2004). No other temperature–morbidity studies have formally investigated the harvesting issue.

*Confounding and modification of the temperature–morbidity relationship.* Some sociodemographic factors might confound and modify the temperature–morbidity relationship. Children and the elderly are usually susceptible to heat- or cold-related health risks. Although there was evidence for heat-related increases in emergency admissions for children < 5 years of age (Kovats et al. 2004), more studies reported the highest-risk age groups to be those > 65 years (Hong et al. 2003; Knowlton et al. 2009; Semenza et al. 1999) or 75 years of age (Johnson et al. 2005; Kovats et al. 2004; Lin et al. 2009). Women have been reported to have a greater risk for coronary events, ACS, and ischemic stroke in cold periods than do men (Barnett et al. 2005; Hong et al. 2003; Panagiotakos et al. 2004). However, emergency transport cases for heat stroke, cardiac insufficiency, hypertension, myocardial infarction, asthma, chronic bronchitis, and pneumonia were greater for males than for females during the summer in Tokyo (Piver et al. 1999; Ye et al. 2001). Lin et al (2009) reported a higher risk of being admitted to hospital for respiratory diseases during the summer in New York for people of Hispanic ethnicity than for those of non-Hispanic ethnicity (6.1% vs. 1.7%), whereas no effect modification by race/ethnicity (e.g., white, black, Hispanic, Asian) or sex was found in the association between mean apparent temperature and hospital admissions for cardiovascular and respiratory diseases in California (Green et al. 2009).

In many locations, concentrations of air pollutants are associated with meteorological conditions. For example, there is usually a higher ozone concentration in summer, as it is a secondary pollutant caused by the reaction of volatile organic compounds, carbon monoxide, and nitrogen dioxide in the presence of sunlight, whereas particulate matter < 10 µm in aerodynamic diameter (PM_10_) peaks during the winter in many places because of the combustion of coal and/or wood for heating. These pollutants are often controlled for when considering the effect of ambient temperature on morbidity (Kovats et al. 2004; Liang et al. 2008; Linares and Diaz 2008; Michelozzi et al. 2009). However, few studies have explored whether exposure to air pollution modifies associations between temperature and morbidity. Ren et al. (2006) reported that PM_10_ significantly modified the relationship between daily minimum temperature and hospital admissions for cardiovascular and respiratory diseases in Brisbane, Australia, with stronger estimated effects of temperature at higher levels of PM_10_. In a multicity European study, ozone did not appear to modify or confound associations between hot temperature and hospital admissions for cardiovascular, cerebrovascular, and respiratory diseases (Michelozzi et al. 2009).

## Conclusions and Recommendations

We identified 40 relevant studies, most conducted in the United States and Europe during the last decade. Some descriptive studies provided early evidence of heat-related morbidity in specific cities during a single heat wave, and research has expanded recently to address the temperature–morbidity relationship in larger and more diverse populations in multiple areas. Although the case-crossover approach has seldom been used (Green et al. 2009; Hong et al. 2003; Kyobutungi et al. 2005), it is expected to be increasingly applied because of its ability to effectively control for individual-level confounding.

A number of well-controlled studies showed that ambient temperature was significantly associated with total and cause-specific morbidities, in which most reported heat effects with only a few reporting cold effects. Several studies found U- or V-shaped exposure–response relationships, with morbidity increasing at both ends of the temperature scale. The majority of studies reported detrimental effects of heat on the same day or up to the following 3 days, and longer cold effects up to a 2- to 3-week lag, with no substantial effects after more than 1 month.

A number of reasons may explain the heterogeneity of results across these studies. First, previous studies covered a wide range of populations in various geographical locations. Besides different demographic characteristics, some domestic and local adaptation factors could influence the direction and magnitude of the effects of ambient temperature on nonfatal health outcomes. For example, Ostro et al. (2010) estimated that the use of air conditioning could significantly reduce the effects of temperature on hospitalizations for multiple diseases, with 0.76% absolute reduction in excess risk of cardiovascular disease for every 10% increase in air conditioner ownership. Second, many temperature indicators have been used to define exposure, including minimum, mean, maximum temperature, diurnal temperature range, apparent temperature, and Humidex. However, which temperature measure is better to predict morbidity remains to be determined. Third, studies have evaluated different measures of morbidity, including general practitioner visits (Hajat et al. 2004; Hajat and Haines 2002), emergency department visits or admissions (Liang et al. 2009; Wang et al. 2009), and hospitalizations (Lin et al. 2009; Michelozzi et al. 2009). They are not mutually exclusive (e.g., a patient visiting an emergency department could be subsequently admitted to the hospital). Emergency is typically considered to be less severe and more acute than hospitalization, which implies that it can catch the effect of temperature change at the early stage. It has been suggested that studies including emergency department visits may yield more valuable information for describing the epidemiology of temperature-related morbidity than a hospitalization-only study (Knowlton et al. 2009). Finally, there were also many methodological differences across studies, including statistical models, study population characteristics (e.g., age and sex), use of lag days (e.g., a single lag and multiple lag), and potential confounders considered.

The IPCC has projected that global mean surface temperature will increase by 1.8–4.0°C (best estimate) by 2100 relative to 1980–1999 (IPCC 2007a). Therefore, efforts to understand how climate change will affect health are urgently needed. Further studies are warranted to determine appropriate measures of exposure for morbidity research; to estimate nonlinear delayed temperature effects; to investigate the threshold temperatures in specific locations; and to understand the relative importance and interactive effects of air pollutants and temperature on morbidity, especially in areas with high air pollution. More multicity studies with consistent methodology should be conducted to make it easy to compare and interpret the temperature effects on morbidity across cities. There is also a need to consider more than one type of morbidity and to track cases from one health service to another by linking medical records. Such studies will provide valuable information for designing and implementing intervention strategies to alleviate the public health impacts of climate change.
